# First person – Madelyn Jackstadt

**DOI:** 10.1242/dmm.049787

**Published:** 2022-08-16

## Abstract

First Person is a series of interviews with the first authors of a selection of papers published in Disease Models & Mechanisms, helping early-career researchers promote themselves alongside their papers. Madelyn Jackstadt is first author on ‘ A multidimensional metabolomics workflow to image distribution and evaluate pharmacodynamics in adult zebrafish’, published in DMM. Madelyn is a PhD student in the lab of Gary Patti at Washington University, St. Louis, MO, USA, and is interested in utilizing metabolomics, particularly isotope tracing, in zebrafish to investigate biological questions including drug effects and disease states.


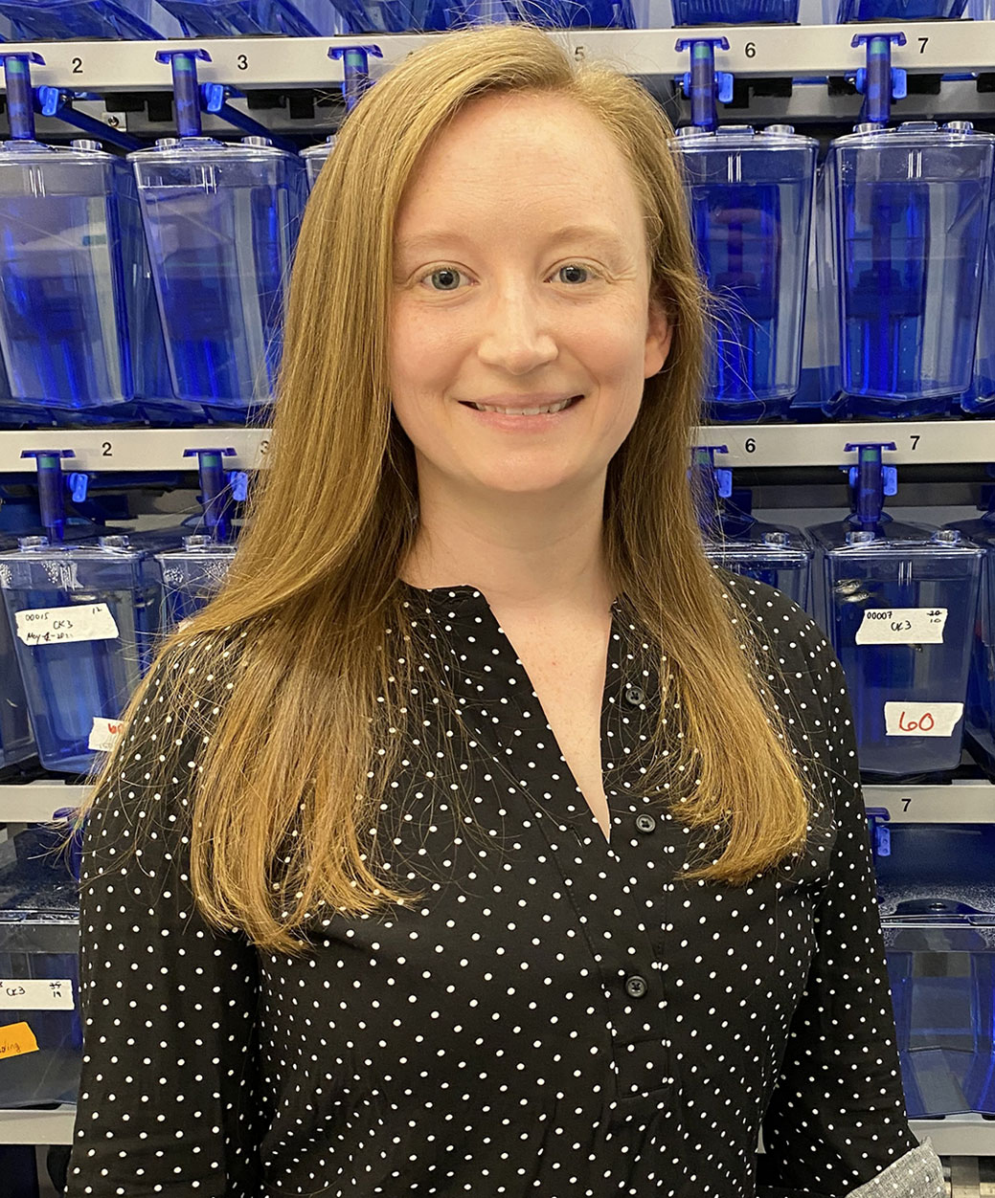


**Madelyn Jackstadt**

**How would you explain the main findings of your paper to non-scientific family and friends?**

For years, pharmaceutical drugs, including hydroxychloroquine sulfate, have been prescribed even though the off-target, or unintended, effects have not been fully characterized. For instance, although a drug might target the liver, it could have unintended consequences in an organ like the brain or heart. Therefore, we could benefit from studies looking at off-target effects from drugs on the entire body. This work demonstrates a pipeline for looking at off-target effects to metabolism in different organs in adult zebrafish. Why zebrafish? Zebrafish are a model organism which have conserved metabolic organs with humans and mice. We can examine changes to metabolism due to drug treatment throughout the animal with images or by comparing the levels of metabolites. Studies using zebrafish for off-target drug effects could help shed light on side effects in humans and add to the pharmaceutical knowledge of drug impact on the entire organism.

“Although a drug might target the liver, it could have unintended consequences in an organ like the brain or heart.”

**What are the potential implications of these results for your field of research?**

The development of this platform allows for researchers to test for drug effects on metabolism and image the biodistribution of drugs throughout the fish. By combining these metabolomics techniques, we found evidence for an off-target metabolic effect in the brain, despite low drug accumulation in this organ shown by both absolute quantitation data and mass spectrometry-based imaging. Although we used a single compound in this study, we note that this workflow could be applied to other water-soluble drugs to examine off-target metabolic effects.

**What are the main advantages and drawbacks of the model system you have used as it relates to the disease you are investigating?**

Zebrafish represent a great model organism for mass spectrometry imaging [including desorption electrospray ionization (DESI) imaging in this work] as you can see the entire animal in a single image, allowing for relative comparisons of drug biodistribution. Adult zebrafish also have organ dimensions which are amenable to liquid chromatography/mass spectrometry analysis of individual organs from individual fish. Finally, water-soluble drugs can be administered directly to the water for fish to uptake. Although zebrafish have conserved metabolic organs with mice and humans, some metabolic pathways are altered. For instance, adult zebrafish do not metabolize excess nitrogen via the urea cycle, so inhibitors of this pathway might not have the full desired effect in this model. Additionally, the platform described in this work utilizes water-soluble drugs, and insoluble compounds would likely have to be administered in another way.

**Figure:**
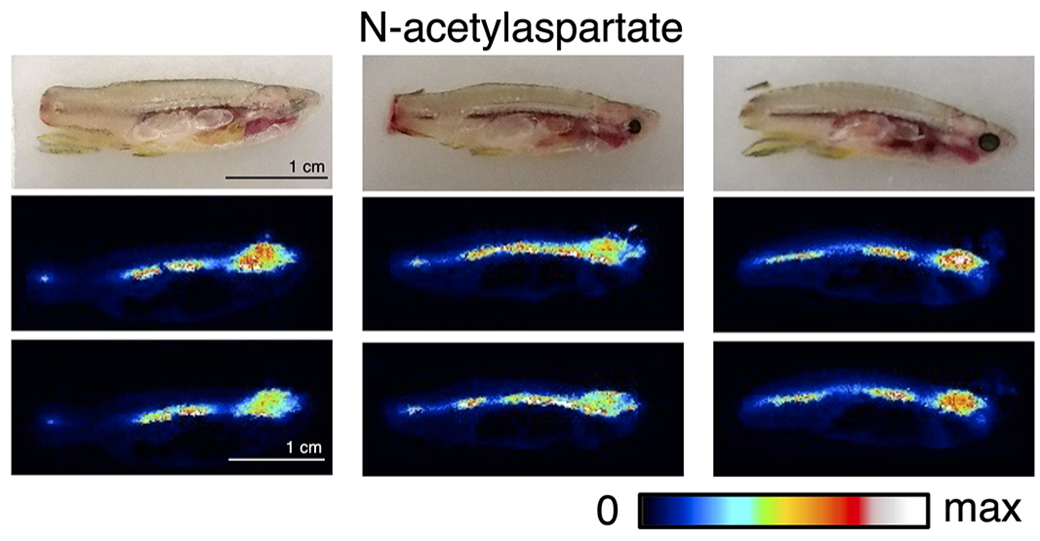
**Localization of N-acetylaspartate to the central nervous system in adult zebrafish as seen with DESI mass spectrometry imaging.** Reference photographs (top) show that areas of high intensity (red/white) in DESI images (below) correspond to the brain and spinal cord, indicating high abundance of N-acetylaspartate in the central nervous system. DESI images are normalized to the total ion current (TIC). Scale bars: 1 cm.

**What do you think is the most significant challenge impacting your research at this time and how will this be addressed over the next 10 years?**

Currently a minus (and a plus!) in the field of zebrafish metabolomics is the limitation of published methods. This is especially true of isotope tracing. This is a plus for those of us who enjoy method development but a minus for researchers newer to the field, especially those without zebrafish or metabolomics backgrounds. Hopefully, over the next ten years, methods like this platform will continue to be developed, utilized and refined.

**What changes do you think could improve the professional lives of early-career scientists?**

From my perspective, early-career researchers could benefit from more resources. In terms of funding, both adequate pay and funding for scientific discovery in the lab should be provided. Additionally, resources for early-career researchers in terms of career planning, mentoring at different stages and information on the hidden curriculum should be offered. A combination of these resources can help retain strong scientists, with less selection bias for those from certain backgrounds and identities.

**What's next for you?**

I am currently working on applying these techniques to look at different biological questions and disease states as part of my PhD research.
